# Deep Learning Fundus Image Analysis for Diabetic Retinopathy and Macular Edema Grading

**DOI:** 10.1038/s41598-019-47181-w

**Published:** 2019-07-24

**Authors:** Jaakko Sahlsten, Joel Jaskari, Jyri Kivinen, Lauri Turunen, Esa Jaanio, Kustaa Hietala, Kimmo Kaski

**Affiliations:** 1 Dept. of Computer Science, Aalto University School of Science, Espoo, 00076 Finland; 2 Digifundus Ltd., Tietotie 2, 90460 Oulunsalo, Finland; 3 Central Finland Central Hospital, Keskussairaalantie 19, 40620 Jyväskylä, Finland

**Keywords:** Medical imaging, Retinal diseases, Machine learning

## Abstract

Diabetes is a globally prevalent disease that can cause visible microvascular complications such as diabetic retinopathy and macular edema in the human eye retina, the images of which are today used for manual disease screening and diagnosis. This labor-intensive task could greatly benefit from automatic detection using deep learning technique. Here we present a deep learning system that identifies referable diabetic retinopathy comparably or better than presented in the previous studies, although we use only a small fraction of images (<1/4) in training but are aided with higher image resolutions. We also provide novel results for five different screening and clinical grading systems for diabetic retinopathy and macular edema classification, including state-of-the-art results for accurately classifying images according to clinical five-grade diabetic retinopathy and for the first time for the four-grade diabetic macular edema scales. These results suggest, that a deep learning system could increase the cost-effectiveness of screening and diagnosis, while attaining higher than recommended performance, and that the system could be applied in clinical examinations requiring finer grading.

## Introduction

Diabetic retinopathy is the most common microvascular complication in diabetes^1^, for the screening of which the retinal imaging is the most widely used method due to its high sensitivity in detecting retinopathy^2^. The evaluation of the severity and degree of retinopathy associated with a person having diabetes, is currently performed by medical experts based on the fundus or retinal images of the patient’s eyes^3^. As the number of patients with diabetes is rapidly increasing, the number of retinal images produced by the screening programmes will also increase, which in turn introduces a large labor-intensive burden on the medical experts as well as cost to the healthcare services. This could be alleviated with an automated system either as support for medical experts’ work or as full diagnosis tool. There are two recent studies that have investigated the use of deep learning systems in automated detection of diabetic retinopathy^4,5^. Both show that an automated system, based on the deep learning artificial neural network approach, can achieve high sensitivity with high specificity in detecting the referable diabetic retinopathy, defined as moderate or worse diabetic retinopathy. There are also other referable eye complications that have recently been investigated with this approach, such as diabetic macular edema^4^, and possible glaucoma and age-related macular degeneration^5^.

For an automated system to be clinically viable, it should be able to classify retinal images based on clinically used severity scales, such as the proposed international clinical diabetic retinopathy and diabetic macular edema disease scales^6^, which are also used in Finland^7^. In the literature one can find recent experiments^8,9^ for the former case of diabetic retinopathy scale, but there are no experiments yet to classify macular changes with the latter scale. Another substantial barrier to broader and more effective use of deep learning system is thought to be the large quantity of annotated images needed for the model to learn^10^.

In this study, our aim is to identify retinopathy using five different diabetic retinopathy and macular edema classification systems. In addition to the earlier studies of referable diabetic retinopathy classification system we also present state-of-the-art results for the clinically used five grade classification and for the first time four grade macular edema classification. Moreover, we present what preprocessing and regularization steps to the images needs to be done for the good functionality of the deep learning system and investigate systematically how the size with much smaller number of images used in training affects its performance.

## Methods

### Original fundus image dataset

The research of present study was done in collaboration with Digifundus Ltd, an ISO 9001:2015 certified provider of diabetic retinopathy screening and monitoring services in Finland. Digifundus Ltd provided a non-open, anonymized retinal image dataset of patients with diabetes, including 41122 graded retinal color images from 14624 patients. The images were taken with Canon CR2 retinal camera after inducing mydriasis with tropicamide 5 mg/ml eyedrops. Two 45 degree color fundus photographs, centered on fovea and optic disc were taken from the patient’s both eyes. The output images were of variable resolutions, ranging from 3888 × 2592 to 5184 × 3456 pixels.

The present study is a methodological study with anonymized medical data and without any intervention in the integrity of a person such as contact with a person. In Finnish law this is not considered as a medical study requiring approval by an ethics committee or a written consent of a person^11^.

### Retinal image grading systems and gradability

Each of the retinal images had been graded with respect to three different criteria, (i) diabetic retinopathy, (ii) macular edema, and (iii) gradability. Images are graded with the proposed international clinical diabetic retinopathy and macular edema disease severity scales^6^, denoted later as PIRC and PIMEC, respectively. The image gradability is a two-stage system, which considers an image to be either gradable or not. All personnel participating in retinopathy assessment had over 10 years’ experience in diabetic retinopathy grading. Retinal images with no lesions or mild diabetic lesions were graded by an optometrist and an M.D. trained for retinopathy grading. All images with moderate or worse changes were graded by two ophthalmologist both with more than 10 years of experience in grading fundus images. If there was a disagreement in grading, such an image was not included in this study.

PIRC and PIMEC grades were further used to obtain additional three types of grading systems: (i) a binary system of *nonreferable*/*referable diabetic retinopathy* (NRDR/RDR), (ii) a binary system of *nonreferable*/*referable diabetic macular edema* (NRDME/RDME), and (iii) three-class system of ungradable/NRDR/RDR. The NRDR/RDR system considers the cases with no diabetic retinopathy and mild diabetic retinopathy as nonreferable diabetic retinopathy, and the cases with moderate or worse diabetic retinopathy as referable diabetic retinopathy. This system has been used in recent works investigating automated detection of diabetic retinopathy^4,5^. The NRDME/RDME system here is defined such that the absence of macular edema is defined as nonreferable diabetic macular edema and any level of macular edema as referable diabetic macular edema. Note that only the gradable images were graded for diabetic retinopathy and macular edema. Ungradable images were included in a single task, in combination with referable diabetic retinopathy classification, which constitutes the grading system QRDR, in which each image is considered to be either ungradable, depicting nonreferable diabetic retinopathy, or depicting referable diabetic retinopathy (ungradable/NRDR/RDR).

### Image preprocessing and dataset division

In the model training and subsequent primary validation, we used preprocessed versions of the original images. The preprocessing consisted of image cropping followed by resizing. Each image was cropped to a square shape which included the most tightly contained circular area of fundus. The procedure removed most of the black borders and all of the patient related annotations from the image data. Each of the cropped images were then resized to five different standard input image sizes of 256 × 256, 299 × 299, 512 × 512, 1024 × 1024, and 2095 × 2095 pixels. The largest image size was the smallest native resolution of the retinal cameras after the preprocessing steps. Here the creation of multiple resolutions was done for the purposes of analyzing the effect of the input image resolution on the classification performance.

The obtained processed datasets were divided into three sets: *training*, *tuning*, and *primary validation* set in the 70%, 10% and 20% proportions of the whole image dataset, respectively, separately for each of the grading systems used in the experiments. In the division per a particular grading system, the different sets were to have similar grade distributions, and that the dataset data per patient to not reside in multiple but only in one of the three sets (of training, tuning, and primary validation), in order to prevent the possibility of obtaining over-optimistic results due to data memorization. Table 1 shows the statistics of the resulting divisions that were used in the experiments. Note that the grade distributions across the different sets were similar, with respect to each grading system, for example, when we consider the NRDR/RDR-system, the proportion of images associated with referable diabetic retinopathy in the training, tuning and primary validation set 44%, 43.9% and 43.4%, respectively.

**Table 1:** Dataset summary.

Grading system	Patients/Label	Training	Tuning	Primary validation
RDR	Patients, No.	8694	1313	2477
	Images Total, No. (%)	24806 (100)	3706 (100)	7118 (100)
	Images Grade 0, No. (%)	13895 (56.0)	2079 (56.1)	4031 (56.6)
	Images Grade 1, No. (%)	10911 (44.0)	1627 (43.9)	3087 (43.4)
PIRC	Patients, No.	8770	1259	2455
	Images Total, No. (%)	24941 (100)	3560 (100)	7129 (100)
	Images Grade 0, No. (%)	11160 (44.7)	1573 (44.2)	3229 (45.3)
	Images Grade 1, No. (%)	2793 (11.2)	408 (11.5)	842 (11.8)
	Images Grade 2, No. (%)	9221 (37.0)	1312 (36.9)	2597 (36.4)
	Images Grade 3, No. (%)	1480 (5.9)	225 (6.3)	382 (5.4)
	Images Grade 4, No. (%)	287 (1.2)	42 (1.2)	79 (1.1)
RDME	Patients, No.	8669	1281	2534
	Images Total, No. (%)	24651 (100)	3675 (100)	7304 (100)
	Images Grade 0, No. (%)	20819 (84.5)	3113 (84.7)	6162 (84.4)
	Images Grade 1, No. (%)	3832 (15.5)	562 (15.3)	1142 (15.6)
PIMEC	Patients, No.	8708	1242	2534
	Images Total, No. (%)	24791 (100)	3535 (100)	7304 (100)
	Images Grade 0, No. (%)	20958 (84.5)	2974 (84.1)	6162 (84.4)
	Images Grade 1, No. (%)	1531 (6.2)	237 (6.7)	465 (6.4)
	Images Grade 2, No. (%)	1566 (6.3)	222 (6.2)	438 (6.0)
	Images Grade 3, No. (%)	736 (3.0)	102 (2.9)	239 (3.3)
QRDR	Patients, No.	10232	1466	2926
	Images Total, No. (%)	28787 (100)	4109 (100)	8226 (100)
	Images Grade 0, No. (%)	3827 (13.3)	533 (13.0)	1132 (13.8)
	Images Grade 1, No. (%)	14005 (48.7)	1991 (48.5)	4009 (48.7)
	Images Grade 2, No. (%)	10955 (38.1)	1585 (38.6)	3085 (37.5)

Class distribution shown on as amounts and percentages the dataset divisions between training, tuning, and primary validation used in experiments.

### Deep learning model

In order to distinguish features related to diabetic retinopathy and macular edema in the color images of patients’ fundi we chose to use a deep convolutional neural network. The neural networks are mathematical models, which consist of parameters used in specific calculations, such as convolutions and summations. Here the neural network can be constructed in such a way, that it receives an input which is used in calculating an output^12^, such as class or grade of diabetic retinopathy. The parameters used in the calculation of the output can be modified in a data-driven manner, by minimizing the error between neural network produced detections of classes and the manual annotations.

The network architecture, we selected, is based on the Inception-v3 architecture^13^ that was pretrained on ImageNet dataset^14^, and is similar to the base model used for the classification of referable diabetic retinopathy by Gulshan *et al*.^4^. Due to the memory constraints, the models trained on 2095 × 2095 pixels input images were trained using mini-batch size of 1, their batch normalization layers were replaced by instance normalization layers, and parameter updates were accumulated across 15 mini-batches. A detailed description of the neural network training and design is presented in the Supplementary Information.

In this study we are basing our classification tasks on a single model for each task and input image resolution or size. However, for the largest input image size there are, apart from the memory constraints, intense computational requirements which translates to long training as well as inference times. For example the training time on our workstation hardware with Graphical Processing Units for 2095 × 2095 pixels input images takes approximately 40 days of consecutive model training, which can be seen impractical. To alleviate this problem of the time budget of training and of inference as well, we have for comparison purposes also employed an ensemble approach of six deep learning models of smaller 512 × 512 pixel input image size, similarly with some previous works^4,5,8^. In the Results section the results for 512 × 512 pixel input size ensemble of six models are presented and compared with the single model results. Further details of the ensemble model approach is included in the Supplementary Information.

### Model evaluation and comparison against previous works

The present study was conducted by training a separate model for each of the five classification tasks and five input image sizes. To evaluate the performance of our model in binary classification tasks we use the receiver operating characteristic (ROC) curve from which we determine the area under the ROC curve (AUC) as well as accuracy, sensitivity, and specificity, while in the multi-class cases we use the area under macro average of ROC (macro-AUC) for each class calculated in one-vs-all manner, accuracy and quadratic-weighted kappa score. Also, we calculate the confusion matrices for the multi-class classification tasks. For each metric in the binary classification tasks, the exact 95% confidence interval (CI) was calculated using the Clopper-Pearson method, similar to that in Gulshan *et al*.^4^ for comparison. In a recent publication^5^ a different confidence interval estimation method was used, namely the cluster-bootstrap, providing a bias-corrected, asymptotic 2-sided 95% CI. This approach used a patient-level clustering for estimating the AUC at the population level. In addition, the models trained for NRDR/RDR were evaluated on the Messidor dataset^14^. The Messidor dataset is labelled for 4-grade grading system for diabetic retinopathy and 3-grade grading system for risk of macular edema. For the evaluation, the Messidor images were labelled as referable if the image had labels for diabetic retinopathy grade ≥2 and/or risk of macular edema grade ≥1, otherwise images were labelled as nonreferable.

We conduct the performance comparison of our model against the performance of the recently presented systems described by Gulshan *et al*.^4^, Ting *et al*.^5^ and in Krause *et al*.^8^. The study by Gulshan *et al*.^4^ using a large set of labeled images, was the first deep learning system achieving high values of sensitivity and specificity in detecting referable diabetic retinopathy. The system developed by Ting *et al*.^5^ for the same task, was trained using large datasets of multi-ethnic population, achieved nearly comparable results to those by Gulshan *et al*.^4^ A more recent work described in Krause *et al*.^8^ extended these works, by focusing on classifying the severity of diabetic retinopathy using the five-level PIRC scale. The present study extends these previous studies: (i) by using significantly less images to train the deep learning system; (ii) by conducting experiments on five different classification systems, including two clinically used scales, PIRC and PIMEC (which has not been investigated by any previous related studies); (iii) by performing training for different tasks without aggregated model prediction to provide independent results for each task; and (iv) by investigating the effect of image resolution to the model, in order to find out their effect on model performance and a trade-off between the number (or cost) of manually annotated images, image resolution and performance.

## Results

In the binary classification tasks, i.e. NRDR/RDR and NRDME/RDME, our algorithm achieved the best results using the largest 2095 × 2095 pixels input image size. In the NRDR/RDR classification on our primary validation set having 7118 images, our algorithm achieved the sensitivity of 0.896 (with 95% CI: 0.885–0.907) and specificity 0.974 (with 95% CI: 0.969–979) and AUC of 0.987 (with 95% CI: 0.984–0.989). Our model performance was evaluated at the operating point where the tuning set achieved 0.900 sensitivity, in a similar manner to Ting *et al*.^5^, while Gulshan *et al*.^4^ had two operating points namely at a high specificity (0.980) point and at a high sensitivity (0.975) point. In Table 2 we present the AUC values of our model, along with the AUC values reported by Gulshan *et al*.^4^ and Ting *et al*.^5^. The Table 2 also illustrates our results and the results reported by Ting *et al*.^4^ at 0.900 sensitivity operating point and results reported by Gulshan *et al*.^4^, closest to the 0.900 sensitivity operating point. Two other recent studies, Krause *et al*.^8^ and Guan *et al*.^9^, also explored the NRDR/RDR classification, but as they do not report results close to the 0.900 sensitivity point, we make a separate comparison with these studies.

**Table 2:** Comparison of classification results for referable diabetic retinopathy.

Author	Train samples	Validation samples	Input size	AUC	Sensitivity	Specificity
Gulshan *et al*.^4^	118419	8788	299	0.991 (0.988–0.993)^a^	0.903 (0.875–0.927)^a^	0.981 (0.978–0.985)^a^
Ting *et al*.^5^	76370	71896	512	0.936 (0.925–0.943)^b^	0.905 (0.873–0.930)^c^	0.916 (0.910–0.922)^c^
Ours	28512	7118	2095	0.987 (0.984–0.989)^a^	0.896 (0.885–0.907)^a^	0.974 (0.969–0.979)^a^

The train and validation samples refer to the image amounts in the respective sets, and the input size refers to the image width and height in pixels. Tuning set is included in the train sample size. Our operating point for sensitivity and specificity is calculated at 0.900 sensitivity for comparison of results at similar operating point to Gulshan *et al*.^4^ and Ting *et al*.^5^.

^a^95% exact CI calculated with Clopper-Pearson method.

^b^95% asymptotic, bias-corrected CI calculated with cluster-bootstrap on patient level.

^c^95% asymptotic CI calculated for each logit with cluster sandwich using on patient level.

**Figure 1:**
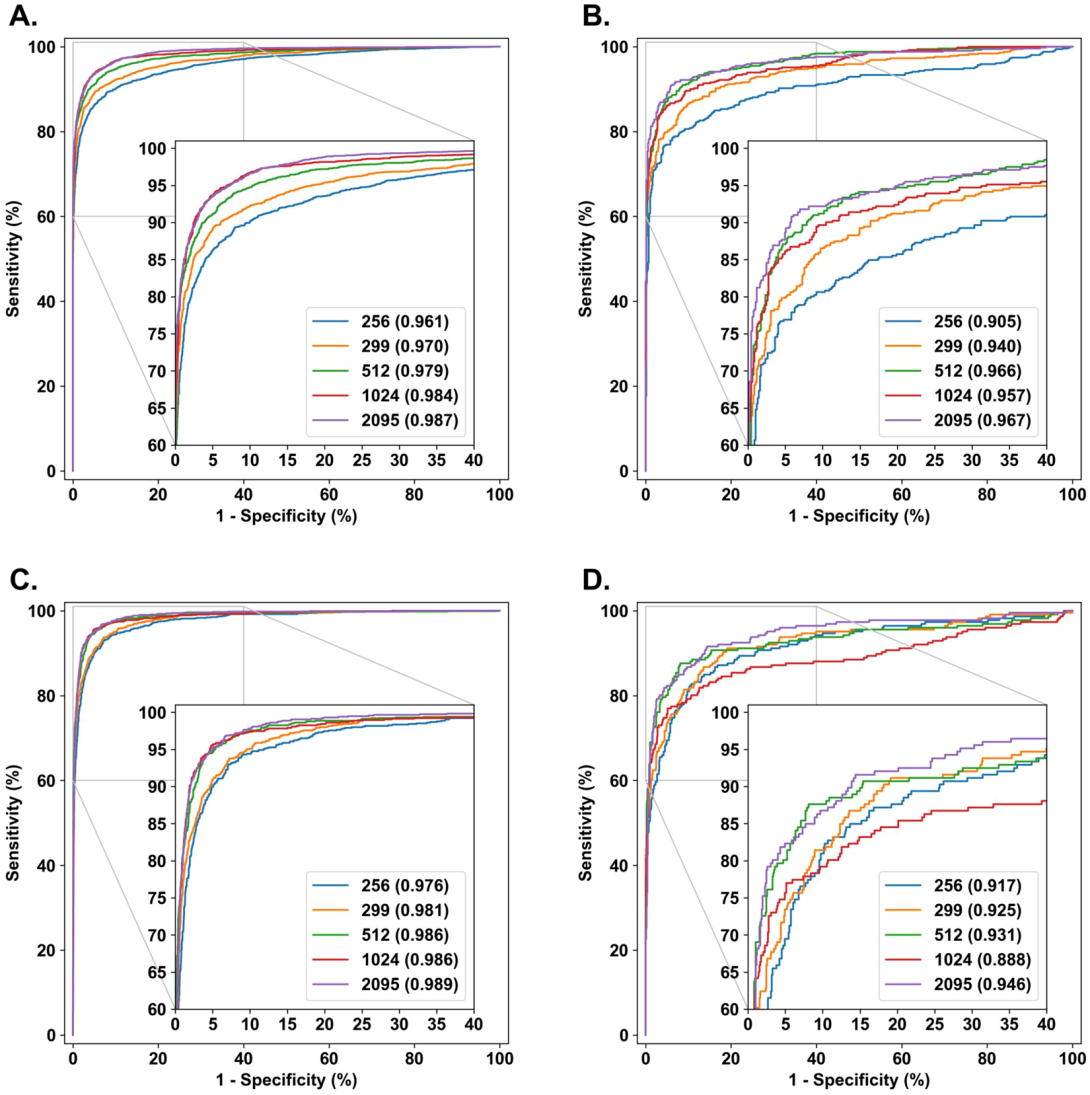
ROC curves for nonreferable vs. referable diabetic retinopathy in classifying nonreferable vs. referable macular edema on primary validation set and Messidor set. (**A)** NRDR/RDR classification on the primary validation set (N = 7118). (**B)** NRDR/RDR classification on Messidor set (N = 1200). (**C)** NRDME/RDME classification on the primary validation set (N = 7304). (**D**) NRDME/RDME classification on Messidor set (N = 1200). Referable vs. nonreferable diabetic retinopathy shown in (**A,B**) and referable diabetic macular edema shown in C and D. ROC curve is shown for input image sizes of 256 × 256, 299 × 299, 512 × 512, 1024 × 1024 and 2095 × 2095 pixels. AUC shown in parentheses in the legend.

As we can see from the Table 2, our result for the AUC, as determined from the ROC curve (see Fig. 1), and the specificity on a similar sensitivity point are on par with the results by Gulshan *et al*.^4^. Our model outperforms the system proposed by Ting *et al*.^5^ in AUC and specificity at the same sensitivity point, 0.900. We have also experimented with a similar operating point selection as Gulshan *et al*.^4^ When operating point was selected for clinical setting, having high specificity of approximately 0.980, our model scored 0.883 (0.880–0.886) sensitivity and 0.980 (0.979–0.981) specificity, in comparison to 0.903 (0.875–0.927) sensitivity and 0.981 (0.978–0.985) specificity reported in Gulshan *et al*.^4^ With the operating point chosen for screening, having high sensitivity, our model scored 0.968 (0.961–0.974) sensitivity and 0.893 (0.883–0.902) specificity, compared to 0.975 (0.958–0.987) sensitivity and 0.934 (0.928–0.940) specificity reported by Gulshan *et al*.^4^

The study presented in Guan *et al*.^9^ also considers the problem of classifying the stage of diabetic retinopathy as nonreferable vs. referable diabetic retinopathy (NRDR/RDR). However, the method they proposed differs considerably from ours, as it models multiple labels, one for each individual labeler (medical expert), whereas our labels are constructed based on agreement between multiple labelers such that each model predicts only one label. In their case the best performing model is trained to predict the PIRC label for an image, and NRDR/RDR label is obtained by aggregating the PIRC grade probabilities. The best results presented in their work include 0.9745 AUC and 0.9093 accuracy for the NRDR/RDR binary classification task, which are both outperformed by our best results of 0.987 AUC and an accuracy of 0.940. They also present 0.8361 specificity at 0.97 sensitivity point, which was outperformed by our model at a similar point with 0.960 sensitivity and 0.909 specificity. The dataset used in Guan *et al*.^9^ was reported to be the same as used by Gulshan *et al*.^4^, however the reported input image size was 587 × 587 pixels.

The more recent study presented in Krause *et al*.^8^ also considers NRDR/RDR classification using aggregated PIRC predictions. They report the AUC of their best model being 0.986, which is comparable to our 0.987 AUC. They also presented the specificity of 0.923 at 0.971 sensitivity, which outperforms our models specificity of 0.909 at 0.960 sensitivity value.

The highest AUC for detecting referable diabetic macular edema (RDME) achieved by our model was 0.989 (0.986–0.991) with 2095 × 2095 resolution images. In Gulshan *et al*.^4^ and Krause *et al*.^8^, the NRDME/RDME classification performance is reported at specified operating points. In Gulshan *et al*.^4^ an operating point with 0.908 (0.861–0.943) sensitivity and 0.987 (0.984–0.990) specificity was reported, whereas at a similar point sensitivity of 0.905 (0.887–0.922) our model had specificity of 0.978 (0.974–0.982), slightly underperforming in comparison. In Krause *et al*.^8^ the sensitivity of 0.949 and specificity of 0.944 were reported for the detection of RDME. Our model achieved 0.954 specificity at similar sensitivity point of 0.947, thus slightly outperforming their proposed system.

In the multiclass classification tasks, i.e. PIRC, PIMEC, and QRDR, our algorithm achieved the best results, in terms of the macro-AUC, using 2095 × 2095 pixels input image size for PIRC and PIMEC tasks and for QRDR best results were achieved with 1024 × 1024 and 2095 × 2095 image sizes. In the PIRC classification task on our primary validation set having 7129 images, our algorithm achieved best performance measured in macro-AUC value of 0.962, with accuracy of 0.869 and quadratic-weighted kappa of 0.910. When the performance was measured using quadratic-weighted kappa, the best performing model was achieved using 1024 × 1024 pixels input image size, with macro-AUC value of 0.961, accuracy of 0.870 and quadratic-weighted kappa of 0.915. Krause *et al*.^8^ reported their system for PIRC classification having the quadratic-weighted kappa value of 0.84, which is outperformed by all our models trained by using larger than 299 × 299 pixels input image size models. Results for multiclass classifications tasks for each image size are shown in Table 3. Additional results for aggregated RDR and RDME predictions are shown in the Supplementary Tables 1 and 2.

**Table 3:** Classification results for PIRC, QRDR and PIMEC with varying input image sizes on the primary validation set.

Grading system	Input size	Macro-AUC	Accuracy	Quadratic-Weighted Kappa
PIRC	256	0.901	0.751	0.772
PIRC	299	0.919	0.785	0.834
PIRC	512	0.951	0.838	0.894
PIRC	1024	0.961	**0.870**	**0.915**
PIRC	2095*	**0.962**	0.869	0.910
PIRC	6 × 512^a^	0.958	**0.944**	0.904
QRDR	256	0.977	0.912	0.901
QRDR	299	0.981	0.922	0.914
QRDR	512	0.989	0.937	0.930
QRDR	1024	**0.991**	**0.938**	**0.932**
QRDR	2095*	**0.991**	0.925	0.914
QRDR	6 × 512^a^	**0.991**	**0.962**	**0.938**
PIMEC	256	0.959	0.928	0.813
PIMEC	299	0.970	0.923	0.803
PIMEC	512	0.979	0.935	0.832
PIMEC	1024	0.978	**0.937**	0.846
PIMEC	2095*	**0.981**	0.934	**0.856**
PIMEC	6 × 512^a^	**0.983**	**0.973**	**0.871**

Macro-AUC refers to area under macro average of ROC for each class one-vs-all manner.

*Trained with model using instance normalization layers and an optimizer with accumulation of 15 mini-batches.

^a^Ensemble of six classifiers trained on same data with same input size.

**Figure 2:**
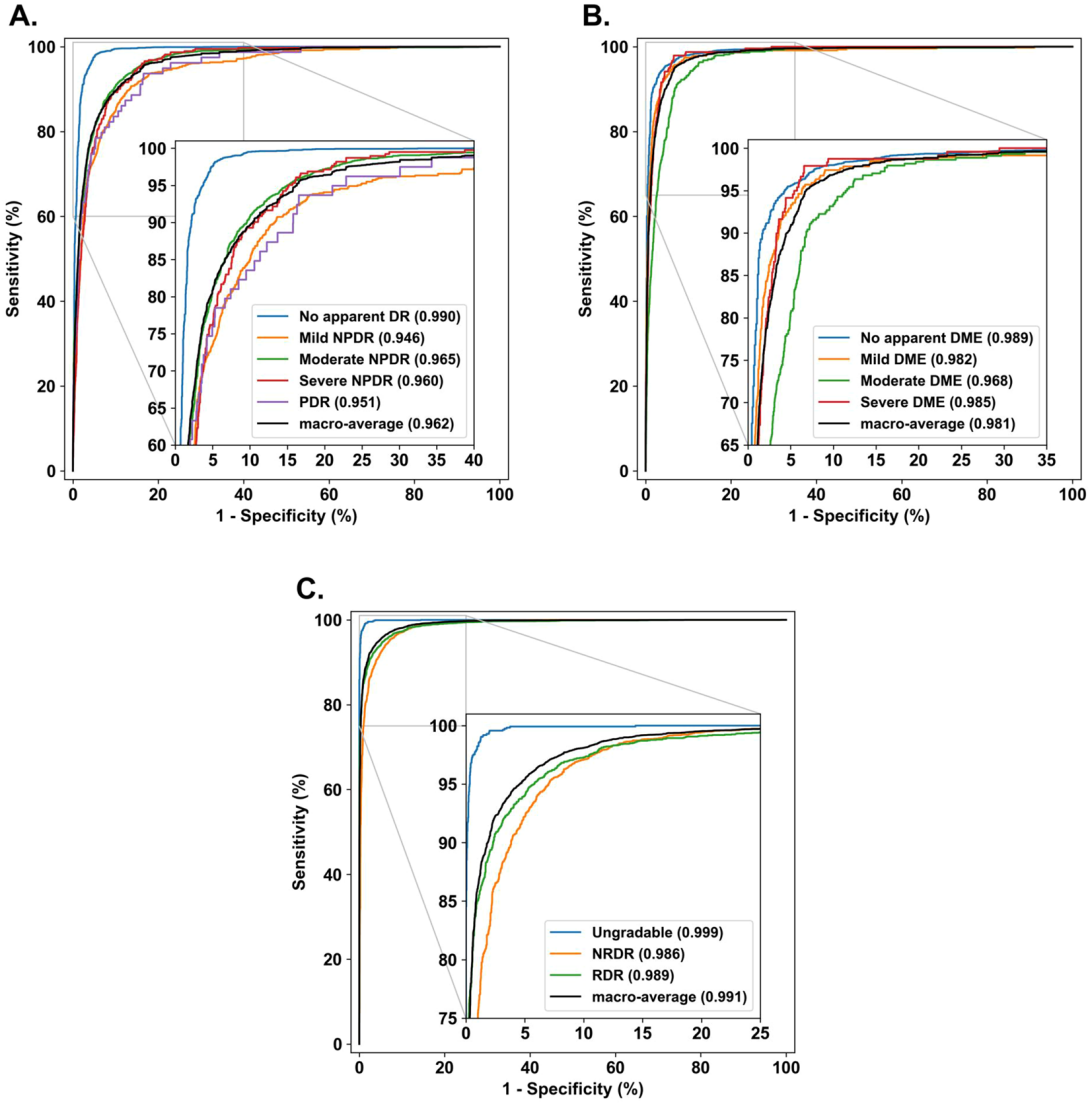
ROC curves for best performing model for each of the multiclass classification tasks. (**A)** PIRC classification on the primary validation set (N = 7129) with input size 2095 × 2095. (**B)** PIMEC classification on the primary validation set (N = 7304) with input size 2095 × 2095. (**C)** QRDR classification on the primary validation set (N = 8226) with input size 1024 × 1024. Multiclass tasks include PIRC, PIMEC and QRDR for the best performing models based on macro-AUC. ROC curves are shown for each class in one-vs-all strategy with addition of macro-average of ROC. Positive class marked in legend with AUC shown in parentheses.

Our experiments also included novel results for QRDR and the clinically used PIMEC grading systems. In the QRDR task on the primary validation set of 8226 images, our algorithm achieved the best results in macro-AUC, accuracy and quadratic-weighted kappa, using the 1024 × 1024 pixels input image size. The model achieved macro-AUC of 0.991, accuracy of 0.938 and quadratic-weighted kappa of 0.932. In the PIMEC classification task the primary validation set of 7304 images, our algorithm achieved the best results for the macro-AUC and quadratic-weighted kappa using 2095 × 2095 resolution images and the best results for accuracy using 1024 × 1024 resolution images. The model with the greatest macro-AUC had the value of 0.981 with accuracy of 0.934 and quadratic-weighted kappa of 0.856. The ROC curves of the best performing models based on macro-AUC are shown in Fig. 2.

The effect of the input image size on the ROC curves in binary classification tasks are illustrated in Fig. 1, in which NRDR/RDR and NRDME/RDME classification systems are considered. From the subfigures and the tables we can see that increasing the input image resolution from 256 × 256 to 512 × 512 clearly improved the results, and even better results were obtained as the resolution was further increased; note that the modeling setup was slightly different for the 2095 × 2095 sized input images than for the others, as mentioned earlier. A similar observation was also made by Krause *et al*.^8^, however their analysis was constrained in the context of PIRC classification, whereas we have systematically analyzed the effect of image resolution for five different classification systems.

In Fig. 1 we provide illustrations of the ROC curves on the Messidor dataset^14^. From these curves we can see that the dependence of the model performance on the input image size is not as clear for the Messidor dataset. This is possibly in part due to the fact that Messidor images have multiple different resolutions, the smallest being of 900 × 900 pixels, which were resized into the sizes shown in Fig. 1. In addition, the imaging equipment used in capturing the Messidor dataset and out primary dataset differ, and it is reported by Decencière *et al*.^15^ that one third of the Messidor images were captured without Tropicamide induced pupil dilation. The detailed numerical results for each input image size on the multi-class and binary classification tasks are presented in Tables 3–5. Confusion matrices for multiclass tasks are shown in Table 6.

**Table 4:** Classification results for predicted of NRDR/RDR and NRDME/RDME with varying input image sizes on the primary validation set.

Grading system	Input size	AUC	Sensitivity	Specificity	Accuracy
RDR	256	0.961	0.895	0.913	0.905
		(0.956–0.965)	(0.884–0.906)	(0.904–0.921)	(0.898–0.912)
RDR	299	0.97	0.896	0.946	0.924
		(0.966–0.974)	(0.884–0.906)	(0.938–0.953)	(0.918–0.930)
RDR	512	0.979	0.9	0.963	0.935
		(0.975–0.982)	(0.888–0.910)	(0.956–0.968)	(0.929–0.941)
RDR	1024	0.984	**0.91**	0.97	**0.944**
		(0.981–0.987)	**(0.899–0.920)**	(0.964–0.975)	**(0.938–0.949)**
RDR	2095*	**0.987**	0.896	**0.974**	0.94
		**(0.984–0.989)**	(0.885–0.907)	**(0.969–0.979)**	(0.935–0.946)
RDR	6 × 512^a^	0.984	0.904	0.971	0.942
		(0.981–0.987)	(0.897–0.911)	(0.967–0.975)	(0.936–0.947)
RDME	256	0.976	0.891	0.953	0.943
		(0.973–0.980)	(0.871–0.908)	(0.948–0.958)	(0.938–0.949)
RDME	299	0.981	0.891	0.96	0.949
		(0.977–0.984)	(0.871–0.908)	(0.955–0.965)	(0.944–0.954)
RDME	512	0.986	0.89	0.976	0.963
		(0.983–0.989)	(0.870–0.907)	(0.972–0.980)	(0.958–0.967)
RDME	1024	0.986	**0.921**	0.974	0.966
		(0.983–0.989)	**(0.904–0.936)**	(0.970–0.978)	(0.961–0.970)
RDME	2095*	**0.989**	0.905	**0.978**	**0.967**
		**(0.986–0.991)**	(0.887–0.922)	**(0.974–0.982)**	**(0.963–0.971)**
RDME	6 × 512^a^	**0.992**	0.904	**0.983**	**0.971**
		**(0.989–0.994)**	(0.897–0.910)	**(0.980–0.986)**	**(0.967–0.974)**

Sensitivity, specificity and accuracy measured at 0.900 sensitivity operating point of tuning set. 95% exact Clopper-Pearson confidence interval in brackets.

*Trained with model using instance normalization layers and an optimizer with accumulation of 15 mini-batches.

^a^Ensemble of six classifiers trained on same data with same input size.

**Table 5:** Classification results for predicted of RDR and RDME with varying input image sizes on the Messidor dataset.

Grading system	Input size	AUC	Sensitivity	Specificity	Accuracy
RDR	256	0.905	0.826	0.872	0.853
		(0.887–0.921)	(0.790–0.858)	(0.845–0.896)	(0.831–0.872)
RDR	299	0.94	0.906	0.824	0.859
		(0.926–0.953)	(0.877–0.930)	(0.794–0.852)	(0.838–0.878)
RDR	512	0.966	**0.945**	0.811	0.868
		(0.954–0.976)	**(0.922–0.963)**	(0.780–0.840)	(0.848–0.887)
RDR	1024*	0.957	0.853	0.955	0.912
		(0.944–0.968)	(0.820–0.883)	(0.937–0.969)	(0.894–0.927)
RDR	2095*^,^**	**0.967**	0.859	**0.971**	**0.923**
		**(0.955–0.976)**	(0.826–0.888)	**(0.956–0.982)**	**(0.907–0.938)**
RDR	6 × 512^a^	0.965	0.92	0.871	0.892
		(0.953–0.974)	(0.903–0.935)	(0.851–0.889)	(0.873–0.909)
RDME	256	0.917	0.619	0.969	0.903
		(0.900–0.932)	(0.553–0.683)	(0.956–0.979)	(0.885–0.919)
RDME	299	0.925	0.633	0.975	0.911
		(0.908–0.939)	(0.566–0.696)	(0.964–0.984)	(0.893–0.926)
RDME	512	0.931	**0.69**	0.989	**0.932**
		(0.915–0.944)	**(0.626–0.750)**	(0.980–0.994)	**(0.917–0.946)**
RDME	1024*	0.888	0.606	0.991	0.918
		(0.869–0.905)	(0.539–0.670)	(0.983–0.996)	(0.901–0.933)
RDME	2095*^,^**	**0.946**	0.597	**0.992**	0.917
		**(0.932–0.958)**	(0.530–0.662)	**(0.984–0.996)**	(0.900–0.932)
RDME	6 × 512^a^	**0.953**	0.575	**0.995**	0.916
		**(0.940–0.965)**	(0.547–0.603)	**(0.989–0.998)**	(0.899–0.931)

Classification on the Messidor set^15^. Sensitivity, specificity and accuracy measured at 0.900 sensitivity operating point of tuning set. 95% exact Clopper-Pearson confidence interval in brackets.

*Messidor images upscaled from input image size of 900 × 900 pixels using bicubic interpolation.

**Trained with model using instance normalization layers and an optimizer with accumulation of 15 mini-batches.

^a^Ensemble of six classifiers trained on same data with same input size.

**Table 6:** Confusion matrices for PIRC, PIMEC and QRDR classification tasks with varying input size on the primary validation set.

Input size	PIRC					PIMEC					QRDR			
**256**	**0**	**1**	**2**	**3**	**4**		**0**	**1**	**2**	**3**		**0**	**1**	**2**
**0**	2982	147	99	0	1	**0**	6053	49	38	22	**0**	1084	45	3
**1**	500	250	92	0	0	**1**	98	332	32	3	**1**	18	3736	255
**2**	343	187	2040	27	0	**2**	80	61	259	38	**2**	0	401	2684
**3**	13	8	279	82	0	**3**	30	10	66	133				
**4**	3	0	51	24	1									
**299**	**0**	**1**	**2**	**3**	**4**		**0**	**1**	**2**	**3**		**0**	**1**	**2**
**0**	3027	142	60	0	0	**0**	5977	79	70	36	**0**	1093	38	1
**1**	438	283	121	0	0	**1**	64	353	42	6	**1**	35	3717	257
**2**	197	198	2128	74	0	**2**	63	45	295	35	**2**	0	311	2774
**3**	6	5	215	154	2	**3**	21	9	95	114				
**4**	4	0	45	25	5									
**512**	**0**	**1**	**2**	**3**	**4**		**0**	**1**	**2**	**3**		**0**	**1**	**2**
**0**	3178	36	15	0	0	**0**	6015	51	78	18	**0**	1108	22	2
**1**	296	440	104	2	0	**1**	60	374	29	2	**1**	50	3786	173
**2**	114	201	2088	193	1	**2**	57	49	317	15	**2**	1	271	2813
**3**	3	3	119	256	1	**3**	19	8	91	121				
**4**	4	0	21	40	14									
**1024**	**0**	**1**	**2**	**3**	**4**		**0**	**1**	**2**	**3**		**0**	**1**	**2**
**0**	3106	95	28	0	0	**0**	6078	45	29	10	**0**	1115	16	1
**1**	149	579	111	0	3	**1**	63	364	36	2	**1**	78	3732	199
**2**	54	195	2257	89	2	**2**	70	55	268	45	**2**	0	218	2867
**3**	4	2	138	222	16	**3**	30	6	67	136				
**4**	4	0	16	18	41									
2095*	**0**	**1**	**2**	**3**	**4**		**0**	**1**	**2**	**3**		**0**	**1**	**2**
**0**	3185	27	16	1	0	**0**	6024	68	51	19	**0**	1069	63	0
**1**	169	517	156	0	0	**1**	42	388	29	6	**1**	22	3926	61
**2**	81	143	2304	69	0	**2**	47	69	262	60	**2**	1	472	2612
**3**	3	1	188	189	1	**3**	14	6	71	148				
**4**	4	0	25	47	3									

Ground truth shown in rows and predicted classes in columns. PIRC classes (0 = no apparent DR, 1 = mild NPDR, 2 = moderate NPDR, 3 = severe NPDR, 4 = PDR), PIMEC classes (0 = no apparent DME, 1 = mild DME, 2 = moderate DME, 3 = severe DME) and QRDR classes (0 = ungradable, 1 = NRDR, 2 = RDR).

*Model trained using instance normalization layers, instead of batch normalization, and optimizer updates accumulated over 15 mini-batches.

In order to investigate the issue of the time budget of training/inference vs. model performance, using an ensemble of smaller input retinal image size models against the single image sized models, we have done additional experiments using an ensemble of six deep learning models with 512 × 512 sized retinal images. These experiments show improved performance in comparison to a single model with the same input image size for all the classification tasks and in some cases improved performance compared to single models using larger retinal input image sizes. On our primary validation set, the ensemble model achieved 0.984 (0.981–0.987) AUC in the task of NRDR/RDR classification being slightly below the best result of 0.987 (0.984–0.989) for the single 2095 × 2095 image size model; 0.992 (0.989–0.994) AUC in the NRDME/RDME classification being slightly above the best result of 0.989 (0.986–0.991) for the single 2095 × 2095 image size model; 0.904 quadratic-weighted kappa in the PIRC classification being slightly below the best result of 0.915 for the single 1024 × 1024 image size model; 0.938 quadratic-weighted kappa in QRDR classification being slightly above the best result of 0.932 for the single 1024 × 1024 image size model and 0.904 quadratic-weighted kappa in PIMEC classification being somewhat above the best result of 0.856 for the single 2095 × 2095 image size model. On the Messidor image dataset, the ensemble model achieved 0.965 (0.953–0.974) AUC in the NRDR/RDR classification being slightly below the best result of 0.967 (0.955–0.976) for the single 2095 × 2095 image size model and 0.953 (0.940–0.965) AUC in the NRDME/RDME classification being slightly above the best result of 0.946 (0.932–0.958) for the single 2095 × 2095 image size model. Further performance measures, analysis and description on the ensemble model are presented in the Supplementary Information. Overall we see that the performance of using an ensemble of six deep learning models with 512 × 512 sized retinal images is more or less the same as the best performance of a single model, usually with larger input image size. However, the main gain of the ensemble-based approach is in the time budget, as training the ensemble of six deep learning models with 512 × 512 sized retinal images took about 102 minutes while training a single model with 2095 × 2095 sized retinal images took about 383 minutes, i.e. nearly four times longer.

The Messidor results reported by Gulshan *et al*.^4^ were obtained on larger Messidor-2 dataset, whereas we use the standard Messidor set, which is a subset of Messidor-2. In addition, it was reported that the Messidor-2 dataset was annotated for NRDR/RDR by Gulshan *et al*.^4^, whereas our labels were derived from the provided Messidor labels to be as close as possible counterparts of the NRDR/RDR system. The labels used in this study are thus not guaranteed to have translated correctly. For the aforementioned reasons, our results on the Messidor dataset are not directly comparable. In addition, it should be noted that our model was trained using only 24% (28512) of the number of images used by Gulshan *et al*.^4^ (118419), 37% of the number of images used by Ting *et al*.^5^ (76370) and only 1.7% of images used by Krause *et al*.^8^ (1662646). However, we trained and evaluated different models using multiple image sizes ranging from 256 × 256 pixels to 2095 × 2095 pixels, whereas the main results reported by Gulshan *et al*.^4^, Ting *et al*.^5^ and Krause *et al*.^8^ were for the image size of 299 × 299 pixels, 512 × 512 pixels and 779 × 779 pixels, respectively. To summarize, the results demonstrate that our proposed system obtained comparable performance to those of the state-of-the-art systems, but by using considerably less or even a small fraction of training images.

## Discussion

In this study, we have presented a systematic computational methodology for diabetic retinopathy and macular edema classification, and assessed its performance on a non-open dataset using five different diabetic retinopathy and macular edema classification systems. We have found that our deep learning model achieved comparable or better results with only a small fraction ( < 1/4) of training set images than used recently by two other groups to obtain the state-of-the-art results in the nonreferable/referable diabetic retinopathy (NRDR/RDR) classification, with similar model architecture. We have also presented state-of-the-art results for classifying retinal images using the proposed international diabetic retinopathy classification system (PIRC), when measured with Cohen’s quadratic-weighted kappa, using less than 2% of the images than previous state-of-the-art system. Our work also sets for the first time the baseline for classifying retinal images using the clinical scale of the proposed international macular edema classification system (PIMEC).

The goodness of these results can most likely be attributed on one hand to regularizing image preprocessing and on the other hand to the features in the dataset and in the experimental setting. For example, our database was prepared with class/grade-balance in mind, so that its grade-distribution when considering the NRDR/RDR classification, was aimed to be uniform and include as many severe cases as possible, thus having a grade distribution which does not necessarily follow a population or a clinical distribution. Other attributing aspects possibly include the fact that the retinal images in our dataset were taken from a rather homogeneous population base in terms of ethnicity; the technical quality and the rate of standardization in the imaging setup within our images, and the quality of their gradings may also have attributed to the goodness of results.

We have also investigated the effect of the size of the images used in training, on the performance of the trained deep learning system in the fundus image classification, an assessment which was limited in previous studies to image sizes less than 779 × 779 pixels, thus excluding near native retinal camera resolutions. In our investigation, the classifier was trained on five different input image sizes, for each of the five classification systems. In all tasks, the best performing model according to AUC/macro-AUC metric was the model with the largest resolution, namely the images of 2095 × 2095 pixels, except that under the QRDR classification the best results were achieved with image sizes of 1024 × 1024 and 2095 × 2095 pixels.

It was observed that the AUC performance overall increased with the input image size, which could be attributed to the fact that the amount of information and features in the images increases with the image size. However, in our implementation, increasing the input image size without modifying the model architecture, dramatically increased the average duration of training and inference as well as memory requirements in the computations. This in fact meant that the modeling setup had to be different for the 2095 × 2095 input images than for the other ones. As an example, training a slightly modified model using the input size of 2095 × 2095 pixels with mini-batch size of 1 increased the average training time for each epoch approximately 20 times in comparison to the base model using an input size of 512 × 512 pixels with the mini-batch size of 6, but with minimal performance improvements in most classification tasks. This suggests that in the case of a fixed wall-clock time requirement for training and inference, it might be better to consider a smaller input image size, and do a wider (grid-)search in terms of the other hyperparameters and/or training an ensemble of classifiers, latter option also suggested by our ensemble model experiment.

Our results on the primary validation dataset using the ensemble of six models trained with 512 × 512 sized retinal images, show overall improved performance in comparison to the single model on same input size, and in the cases of NRDME/RDME, QRDR and PIMEC also in comparison to the single model results using largest 2095 × 2095 sized retinal images, yet with the huge savings in the time budget of training/inference, i.e. reaching comparable performance with nearly one quarter of the wall clock time. However, the computational cost in training an ensemble model grows linearly with the number of models. For smaller than 512 × 512 sized retinal images it is natural to assume on the basis of the single model results that with the ensemble approach savings in the time budget of training/inference will increase even more but with poorer performance. The situation with ensemble approach for larger than 512 × 512 sized images is expected to be the opposite, i.e. giving rise to comparable or even larger time budget of training/inference but with only very marginal improvement to already very good performance of the single models. It is interesting to note that deep learning models trained on a small set of retinal images do not learn identical features, as there exists variation in the predictive performance between the single model and the ensemble model trained on same sized images, even when the neural networks are identically initialized to ImageNet pretrained weights, which is a valuable observation. Furthermore, we can state that the ensemble approach increases the robustness of the classification, as evidenced by our ensemble model experiment outperforming the single 512 × 512 retinal image based model in every classification task. These observations are of practical importance from the deep learning methodology and medical technology synthesis points of view.

Finally, we acknowledge the following limitations of our present deep learning AI-system. The first one concerns the image grading reference, which in our case was provided as an agreement of two experienced and qualified graders for each image, but could unavoidably include grader biases that can result in decreased generalization performance of the model. In addition, as was expressed in Gulshan *et al*.^4^, deep learning neural networks have an inherent limitation of possibly learning features that are unknown or ignored by medical experts, when the network is only fed in an image and its grading without defining diagnostically important features such as *microaneurysms* and *exudates* as well as their numbers that are important biomarkers of diabetic retinopathy.

In our study we have demonstrated that a deep learning AI-system applied to a relatively small retinal image dataset could accurately identify the severity grades of diabetic retinopathy and macular edema and that its accuracy was improved by using high resolution and quality images.

## Supplementary information

Supplementary Information

## Data Availability

Datasets used in model training, tuning and primary validation were provided by Digifundus Ltd. This dataset is not publicly available and restriction apply to their use. The Messidor dataset may be requested from http://www.adcis.net/en/Download-Third-Party/Messidor.html.

## Code Availability

Code used for preprocessing and deep learning includes proprietary parts and cannot be released publicly. However, preprocessing and deep learning algorithms can be replicated using the information in the Methods section and on the Supplementary Information.
