# Supplementary material for: Deep Learning Fundus Image Analysis for Diabetic Retinopathy and Macular Edema Grading
Source: Sci Rep. 2019 Jul 24;9:10750. doi: 10.1038/s41598-019-47181-w (PMC6656880; doi:10.1038/s41598-019-47181-w)
Supplement: Supplementary file 1 — Supplementary Information [file 41598_2019_47181_MOESM1_ESM.pdf]

Deep Learning Fundus Image Analysis for Diabetic Retinopathy and Macular Edema Grading

Jaakko Sahlsten[1], Joel Jaskari[1], Jyri Kivinen[1], Lauri Turunen[2], Esa Jaanio[2], Kustaa Hietala[3] & Kimmo Kaski[1,*]

February 18, 2019

[1]Dept. of Computer Science, Aalto University School of Science, 00076, Finland. [2]Digifundus Ltd., Tietotie 2, 90460 Oulunsalo, Finland. [3]Central Finland Central Hospital, Keskussairaalantie 19, 40620 Jyväskylä, Finland. [*]Corresponding author: e-mail: kimmo.kaski@aalto.fi, regular mail: P.O. Box 15500, FI-00076 AALTO, Finland.

Supplementary Information

Experimental setup

Initially the network architecture was that of the Inception-v3 optimized for ImageNet dataset[1] classification, but with the exception that the fully-connected layer of the neural network model was replaced with two consecutive fully-connected layers, the former utilizing a regularization technique called dropout and the latter a vanilla fully-connected layer with softmax nonlinearity to define the diabetic retinopathy grade probabilities. Values of all other model parameters than those of the added fully-connected layers were initialized to the parameter values of the model (pre)trained on the ImageNet dataset, and all of the parameters were updated during the training.

Network parameters were fine-tuned with the Adam algorithm[2], also called Adam optimizer, on input image sizes of 2095 × 2095, 1024 × 1024, 512 × 512, 299 × 299, and 256 × 256 pixels. The hyperparameters including the learning rate, dropout rate and mini-batch size were tuned on the tuning set.

The networks using the input image sizes of 2095 × 2095 pixels were defined and trained using minor modifications to the training procedure and to the network architecture. They were trained with the mini-batch size 1, due to memory restrictions of Graphical Processing Units (GPUs) used deep learning neural network computations. The canonical model and optimizer were modified to take deviation into consideration as follows: the batch normalization layers were replaced with instance normalization and the optimizer updates were accumulated and averaged to attain similar updates as with larger mini-batch size. The networks for the input image size of 2095 × 2095 pixels were also trained only with the best guess estimates of the appropriate hyperparameter values due to time restrictions.

The early stopping approach was used in the hyperparameter search with the stopping criterion chosen as the area under the receiver operating characteristic curve (AUC) on a binary classification task and the area under the average of the receiver operating characteristic curves of each class in one-to-all manner (macro-AUC) on a multi-class classification task. In addition, the learning rate was set to decay exponentially so that the learning rate at an epoch $\tau \in [2, . . .]$ was $0.99^{\tau-1}$ times the learning rate at the first epoch ($\tau = 1$), the initial learning rate.

Model prediction aggregation results for RDR and RDME from model's trained on PIRC and PIMEC, respectively, are shown for the primary validation set in Table 1 and for Messidor dataset in Table 2. Aggregated class probabilities are calculated as sum over corresponding model class probabilities using PIRC to NRDR/RDR and PIMEC to NRDME/RDME mappings described in the main text.

We have also employed ensemble model training with 512 × 512 image size. An ensemble of six models were trained on each classification task with the same training data and ImageNet initialization. The ensemble model evaluation on the primary validation and, when applicable on the Messidor set, can be seen in the Supplementary Tables 3 and 4 for binary and multiclass cases, respectively. The results show that the ensemble of six models outperforms the corresponding single model in every experiment with 512 × 512 image size. In addition, the ensemble model results for 512 × 512 image size turn out to be slightly better than our results for larger image sizes (i.e. 1024 × 1024 and 2095 × 2095) in QRDR, PIMEC and RDME classification tasks when measured by AUC for binary classifications and Quadratic-Weighted Kappa for multiclass classifications. As all our models, including the single deep neural networks in the ensemble model have been initialized to ImageNet pretrained Inception-v3 model, the variation in the performance of a single model and the ensemble model cannot be explained by variation caused by random initialization. However, our training procedure can cause variation in the results. We employ dropout regularization method, as described in the Experimental setup of this Supplement, which causes randomness in the training of the models. We also feed the training images in different (randomized) order and with different random augmentations for each of the models trained for the ensemble to encourage the networks to be dissimilar. Different random behavior was ensured by augmenting the random number generator for each model training. The results suggest that even when trained on small number of same retinal images, classifiers can learn different discriminative features based on randomness in training and regularization. The ensemble model class probabilities are derived from the individual model outputs as arithmetic mean of each softmax output over the models.

In this study the models were trained using Nvidia GPU-system, GTX 1080 Ti with 11GB of VRAM, and deep learning frameworks TensorFlow and Keras. The average duration of epoch with 512 × 512 image size and mini-batch size 6 turned out to be 17 minutes, with an ensemble of six using the same settings the average duration was 102 minutes while with 2095 × 2095 image size using instance normalization and mini-batch size of 1 the duration was 383 minutes.

Table 1: Classification results for model trained on PIRC/PIMEC and predictions with aggregated RDR/RDME scores, respectively, with varying input image sizes on the primary validation dataset.

Grading system	Input size	AUC	Sensitivity	Specificity	Accuracy
RDR	256	0.954 (0.949-0.959)	0.878 (0.870-0.885)	0.900 (0.893-0.907)	0.890 (0.883-0.898)
RDR	299	0.971 (0.967-0.975)	0.872 (0.864-0.879)	0.953 (0.947-0.957)	0.918 (0.911-0.924)
RDR	512	0.983 (0.980-0.986)	**0.888 (0.881-0.896)**	0.973 (0.969-0.976)	0.937 (0.931-0.942)
RDR	1024	0.986 (0.983-0.988)	0.886 (0.879-0.893)	0.980 (0.976-0.983)	**0.940 (0.934-0.945)**
RDR	2095*	**0.987 (0.984-0.990)**	0.872 (0.864-0.879)	**0.981 (0.977-0.984)**	0.934 (0.928-0.940)
RDME	256	0.973 (0.969-0.976)	0.906 (0.899-0.913)	0.952 (0.947-0.957)	0.945 (0.939-0.950)
RDME	299	0.982 (0.979-0.985)	0.891 (0.883-0.898)	0.961 (0.957-0.966)	0.950 (0.945-0.955)
RDME	512	0.986 (0.983-0.989)	0.899 (0.892-0.906)	0.972 (0.968-0.975)	0.960 (0.956-0.965)
RDME	1024	0.988 (0.985-0.990)	**0.915 (0.908-0.921)**	0.975 (0.971-0.979)	0.966 (0.961-0.970)
RDME	2095*	**0.989 (0.986-0.991)**	0.884 (0.877-0.892)	**0.984 (0.981-0.987)**	**0.968 (0.964-0.972)**

Sensitivity, specificity and accuracy measured at 0.900 sensitivity operating point of tuning set. 95% exact Clopper-Pearson confidence interval in brackets. 'input size' refers to the heights and widths of the input images in pixels.

* Trained with model using instance normalization layers and an optimizer with accumulation of 15 mini-batches.

Table 2: Classification results for model trained on PIRC/PIMEC and predictions with aggregated RDR/RDME scores, respectively, with varying input image sizes on the Messidor dataset.

Grading system	Input size	AUC	Sensitivity	Specificity	Accuracy
RDR	256	0.924 (0.908-0.938)	**0.941 (0.926-0.954)**	0.467 (0.439-0.496)	0.669 (0.642-0.696)
RDR	299	0.947 (0.933-0.959)	0.900 (0.882-0.917)	0.871 (0.851-0.889)	0.883 (0.864-0.901)
RDR	512	**0.977 (0.966-0.984)**	0.902 (0.884-0.918)	0.955 (0.942-0.966)	**0.932 (0.917-0.946)**
RDR	1024*	0.938 (0.923-0.951)	0.734 (0.708-0.759)	**0.985 (0.977-0.991)**	0.878 (0.858-0.896)
RDR	2095*,**	0.960 (0.948-0.971)	0.800 (0.777-0.823)	0.974 (0.963-0.982)	0.900 (0.882-0.916)
RDME	256	0.866 (0.845-0.884)	0.456 (0.427-0.484)	0.986 (0.977-0.992)	0.886 (0.866-0.903)
RDME	299	0.935 (0.920-0.949)	**0.695 (0.668-0.721)**	0.951 (0.937-0.962)	0.902 (0.884-0.919)
RDME	512	0.950 (0.936-0.962)	0.611 (0.582-0.638)	0.993 (0.986-0.997)	0.921 (0.904-0.935)
RDME	1024*	0.936 (0.920-0.949)	0.597 (0.569-0.625)	**0.994 (0.988-0.997)**	0.919 (0.902-0.934)
RDME	2095*,**	**0.959 (0.946-0.970)**	0.624 (0.596-0.651)	0.992 (0.985-0.996)	**0.922 (0.906-0.937)**

Classification on the Messidor set[3]. Sensitivity, specificity and accuracy measured at 0.900 sensitivity operating point of tuning set. 95% exact Clopper-Pearson confidence interval in brackets. 'input size' refers to the heights and widths of the input images in pixels.

* Messidor images upscaled from input image size of 900 × 900 pixels using bicubic interpolation

** Trained with model using instance normalization layers and an optimizer with accumulation of 15 mini-batches.

Table 3: Classification results for the ensemble model trained on 512 × 512 sized RDR or RDME images. Evaluated on the primary validation set and the Messidor dataset.

Grading system	Dataset	AUC	Sensitivity	Specificity	Accuracy
RDR	Primary validation	0.984 (0.981-0.987)	0.904 (0.897-0.911)	0.971 (0.967-0.975)	0.942 (0.936-0.947)
RDR	Messidor	0.965 (0.953-0.974)	0.920 (0.903-0.935)	0.871 (0.851-0.889)	0.892 (0.873-0.909)
RDME	Primary validation	0.992 (0.989-0.994)	0.904 (0.897-0.910)	0.983 (0.980-0.986)	0.971 (0.967-0.974)
RDME	Messidor	0.953 (0.940-0.965)	0.575 (0.547-0.603)	0.995 (0.989-0.998)	0.916 (0.899-0.931)

Classification on the Messidor set[3]. Sensitivity, specificity and accuracy measured at 0.900 sensitivity operating point of tuning set. 95% exact Clopper-Pearson confidence interval in brackets.

Table 4: Classification results for the ensemble model trained on 512 × 512 sized PIRC, QRDR or PIMEC images. Evaluated on the primary validation set.

Grading system	Macro-AUC	Accuracy	Quadratic-Weighted Kappa
PIRC	0.958	0.944	0.904
QRDR	0.991	0.962	0.938
PIMEC	0.983	0.973	0.871

Supplementary References

1. Russakovsky O, Deng J, Su H, et al. ImageNet Large Scale Visual Recognition Challenge. 2015;115(3):211-252.
2. Kingma DP, Ba J. Adam: A Method for Stochastic Optimization. arXiv e-prints. 2014. https://ui.adsabs.harvard.edu/\#abs/2014arXiv1412.6980K. Accessed December 01, 2014.
3. Decencière E, Zhang X, Cazuguel G, et al. *Feedback on a publicly distributed image database: The Messidor database.* Vol 02014.
